# Is the ^18^F-choline PET-CT more cost-effective than standard protocol for locating a parathyroid adenoma?

**DOI:** 10.1007/s00423-025-03824-3

**Published:** 2025-07-31

**Authors:** Joaquín Ortega-Serrano, Santiago Serrano-López, Raquel Alfonso-Ballester, Rosa Martí-Fernández, María Lapeña-Rodríguez, Rafael Díaz Expósito, Norberto Cassinello-Fernández

**Affiliations:** 1 Department of Surgery, University of Valencia, Valencia, Spain; 2 Unit of Endocrine and Bariatric Surgery, University Clinic Hospital of Valencia, Valencia, Spain; 3 Department of Nuclear Medicine, University Clinic Hospital of Valencia, Valencia, Spain; 4 Department of Surgery, Faculty of Medicine and Odontology, Av. Blasco Ibanez, 15, Valencia, 46010 Spain

**Keywords:** 18F-Choline PET-CT, Primary hyperparathyroidism, Parathyroid adenoma, Selective parathyroidectomy, Minimally invasive parathyroidectomy.

## Abstract

**Purpose:**

The most common cause (> 80% of cases) of primary hyperparathyroidism (PHPT) is parathyroid adenoma. Its diagnosis is conventionally made by cervical ultrasound and ^99m^Tc-MIBI scintigraphy. However [^18^F-Choline PET-CT ( [^18^F-FCh PET-CT) offers greater sensitivity and specificity, although at a high cost, which prevents it from being a first-line diagnostic method.

**Methods:**

Observational retrospective cohort study of 100 consecutive patients operated on for PHPT by parathyroidectomy in a tertiary hospital. Patients were divided into two groups: Group 1, patients with successful diagnosis using conventional tests (42 patients) and Group 2, patients with an initial failed diagnosis who required ^18^F-FCh PET-CT (52 patients). A group with an ideal diagnostic strategy using only ^18^F-FCh PET-CT was simulated and the costs were compared with the groups in the sample.

**Results:**

The sample finally analyzed 94 patients, 78.7% female, mean age 61.73 years. 55,3 % of the patients required a ^18^F-FCh PET-CT for the location diagnosis. The group 2 required more consultations, more complementary tests and a longer interval between the first consultation and the intervention. The ideal diagnostic strategy (€1,399.77/patient) represents a lower cost than the other strategy (€1,730.61/patient).

**Conclusion:**

The diagnosis of location of a parathyroid adenoma with ^18^F-FCh PET-CT required fewer complementary tests and consultations, reducing the interval until surgical intervention, with no difference in surgical results. The costs if ^18^F-Ch PET-CT is performed as the only location diagnostic test are lower when a group of patients is studied, so its use is recommended as a first line diagnostic tool.

**Clinical trial number**: Not applicable.

## Introduction

Primary hyperparathyroidism (PHPT) is the most identifiable cause of hypercalcemia in up to 90% of cases [1].

The only definitive treatment of PHPT is parathyroidectomy of the affected parathyroid gland(s). Regarding the surgical technique, there are two options: minimally invasive parathyroidectomy (MIP) and bilateral cervical exploration (BCE) [2]. To perform MIP, it is necessary to know the location of the pathological gland preoperatively, so the objectives of the imaging tests are to locate the dysfunctional parathyroid gland, whether in its normal or ectopic position [2].

The current conventional diagnosis of location is by simple ultrasound and ^99m^Tc-MIBI scintigraphy. However, there are other tests that can be carried out such as four-dimensional computerized tomography (4D CT), four-dimensional magnetic resonance imaging (4D MRI), and positron emission tomography (PET) with Fluoro-Choline (^18^F-FCh PET-CT), associated or not with CT or MRI [2].

PET is a highly specific imaging technique for metabolic or biochemical activity ^18^F-FCh PET is a molecular imaging test that takes advantage of the accelerated metabolism of phospholipids present in hyperfunctioning parathyroid tissue. The PET-CT combination allows obtaining high-resolution 3D images that support diagnosis and treatment in a more precise way [3].

The ^18^F-FCh radiotracer, compared to the most used single-photon radiotracer (^99m^Tc-MIBI), improves resolution, has a greater capacity to detect multifocal disease, does not require multiphase acquisition or dual scintigraphy of the thyroid and parathyroid [4, 5] and is positive in many cases with negative previous scintigraphy and ultrasound [6]. The main limitation of this test is its high cost and lower availability in many centers. This is why it is often considered a second line imaging technique [2, 7].

A recent meta-analysis has shown that the ^18^F-FCh PET-CT has a sensitivity of 93.8% and a PPV of 97% [8], but currently, it is usually used as a second line test in cases with a negative or inconclusive result after the image taken by ^99m^Tc-MIBI SPECT-CT [2, 9].

The recent practice guidelines of the European Association of Nuclear Medicine value 18 F-FCh PET-CT in the diagnosis and localization of parathyroid adenomas, and even in cases of secondary hyperparathyroidism, not only due to its higher resolution but also due to its lower irradiation and shorter acquisition time. They also mention the possibility of combining it with MRI, a method that requires less irradiation than CT. Its drawbacks include the possibility of false positives in thyroid inflammation or lymphadenopathy. Among the drawbacks, they mention too its higher price and the lack of cost-effectiveness studies [10, 11]. However, after the publication of these guidelines, several studies have been published in this regard [12–14].

Some of these studies have a theoretical basis [12, 13] and we wanted to study the reality of the diagnostic process in our setting, evaluating the costs generated in our hospital by patients treated for primary hyperparathyroidism caused by an adenoma. Clearly, in an isolated case, the conventional strategy is cheaper and can be just as effective as that using ^18^F-FCh PET/CT. However, it is possible that, by globally evaluating all patients operated on for PHPT, the diagnostic location process may be more efficient using ^18^F-FCh PET/CT as the first and only test, so we believe that it is justified to study whether a change in strategy can also be justified from an economic point of view.

## Materials and methods

A cohort study was carried out on the last 100 consecutive patients operated on for PHPT in the General Surgery Department of our hospital. The only criterion for inclusion was to have undergone a parathyroidectomy, with at least one year of postoperative follow-up. The interventions were performed between the years 2021 and 2023. Causes for exclusion were patients in whom a bilateral cervical examination was performed, or in whom a thyroidectomy was added. This was done to exclude the bias in a study aimed at evaluating the location of a single adenoma, since in those cases the duration and complications of surgery may vary.

This research was carried out in accordance with the tenets of the Declaration of Helsinki. This is an observational retrospective study on an anonymized database, without any intervention on the patients. The Research Ethics Committee (CEIm) of the University Clinic Hospital of Valencia confirmed that no ethical approval was required. All patients signed an informed consent which includes the use of data for scientific studies.

In the patients, a conventional protocol was carried out for the diagnosis of the location of the parathyroid adenoma, consisting of a visit to the Outpatient Clinics of the Endocrinology Department, with the performance of a high-resolution cervical ultrasound and a scintigraphy or a SPECT with ^99m^Tc-MIBI. When the two examinations have been positive and coincident, it is considered located and is transferred to the Surgery Department. If both or any of the examinations are negative or not clear, with doubtful or not consistent results, a ^18^F-FCh PET-CT is requested, and once located, and after the visit to the Outpatient Surgery Clinic, the surgical intervention is scheduled.

To calculate the cost of the conventional protocol, all the consultations and tests performed on the patients in the study population have been collected, from the time of the first visit to the hospital, until the time of discharge.

Of the total number of patients, two groups have been differentiated:

- Group 1: Patients with a successful initial conventional diagnosis, in whom a ^18^F-FCh PET-CT was not performed.
- Group 2: Patients with an initial failed or uncertain diagnosis, in whom a ^18^F-FCh PET-CT was performed.

To meet the objectives of the work, the following parameters have been collected and compared between both groups:

- Age and sex ratio.
- Serum levels of Ca, P and PTH, pre- and post-operative.
- Number of tests and consultations performed.
- Number of days between the first visit and surgery.
- Duration of surgery.
- Intra- and post-operative complications.
- Results of surgery.

To meet the main objective of the work, the costs of the preoperative tests and consultations of all patients have been taken and compared with the costs of an ideal protocol, in which ^18^F-FCh PET-CT would be performed as the only diagnostic test, and which would consist of the following steps:

- A first visit to Endocrinology, with request for analytical values ​​of Ca, P and PTH, and request for ^18^F-FCh PET-CT.
- A subsequent visit to Endocrinology, to review the test results and refer to Surgery.
- A first visit to Surgery, to indicate the intervention, request the preoperative protocol and include the patient on the surgical waiting list.
- A subsequent visit to Surgery, to see the results of the preoperative tests and schedule the intervention.

The cervical ultrasound was performed in the Outpatient Clinic by the doctors of the General Surgery or the Radiodiagnosis and Imaging Departments of the hospital itself. The ^99m^Tc-MIBI scintigraphy/SPECT was performed in the Nuclear Medicine Department. A double phase scintigraphic study was carried out. A dose of 20 ± 5 mCi of ^99m^Tc-MIBI was injected and at 15- and 90-minutes, images were taken in the cervicothoracic region. In most cases, it was complemented by SPECT, less than 60 min after the injection of the radiotracer. The ^18^F-FCh PET-CT was performed in the Nuclear Medicine Department of the hospital, or in complementary facilities outside the center. 5 ± 2 mCi of ^18^F-FCh were administered intravenously and 60 min later a PET-CT study was performed in the cervicothoracic region. In addition, 3 min after the initial study, a low-dose CT was performed to correct the attenuation.

All patients underwent minimally invasive selective parathyroidectomy.

To assess the results of the surgery, serum calcium, phosphorus and PTH were determined at preoperative, intraoperative and postoperative levels.

The diagnostic tests have been valued taking into account the price of each test in the Generalitat Valenciana Fees Law for the year 2022 [15]. The value of the consultations has been taken from the Hospital’s Economic Information System (EIS) for the year 2022 (Table 1) [16]. Only the cost data relating to the preoperative period have been compared, given that the objective of the study is to differentiate diagnostic protocols for location, the protocol being identical from the surgical intervention onwards.

**Table 1:** Prices of the tests and consultations

Consultation/Test	€ Price
1 st Consultation Endocrinology	152.26
Subsequent Consultation Endocrinology	76.13
1 st Consultation Surgery	41.04
Subsequent Consultation Surgery	20.52
Cervical ultrasound	65.35
Cervical CT with EV contrast	155
^99m^Tc-MIBI gammagraphy/SPECT	133,25
^18^F-FCh PET-CT	1,026.66
Blood tests	23.16
Ca	0.63
P	0.62
PTH	21.91

The cost of the different diagnostic strategies was calculated by adding the cost of the pre-surgical endocrinology and general surgery consultations, the diagnostic imaging techniques used for the diagnosis of location and the preoperative blood tests. It should be noted that the costs shown in this work do not refer to the total cost of the diagnostic-therapeutic process of the patients, but to the set of tests that would be different if the ^18^F-FCh PET-CT were used as the first-line diagnostic process.

A descriptive study of the results was carried out. Continuous variables are expressed as mean values ​​with standard deviation, except for the number of imaging tests, which are expressed as medians and ranges. Categorical variables are expressed as a percentage of the total. A cost analysis was performed comparing groups 1 and 2 and the ideal proposed diagnostic strategy for parathyroid adenoma. To study the differences in quantitative variables between the two groups (successful conventional diagnosis and failed conventional diagnosis), a t-Student test was performed for each variable, considering differences as statistically significant for values ​​of *p* < 0.05.

Statistical analysis of the data was performed with IBM SPSS Statistics version 25.0 (IBM Corp., Amonk, NY, USA).

## Results

Between March 2021 and July 2023, 100 patients operated on with PHPT underwent the conventional strategy for location diagnosis of pathological glands using ^99m^Tc-MIBI scintigraphy/SPECT and/or cervical ultrasound, adding, if necessary ^18^F-FCh PET-CT. From the initial sample, 6 patients were excluded: 4 patients with a multiglandular disease, in which BCE was performed, and 2 patients in whom a hemithyroidectomy was also performed, so 94 patients were definitively included in this study.

Of the patients included in the study, 42 underwent the conventional diagnostic strategy (Group 1) and 52 underwent ^18^F-FCh PET-CT in addition to the conventional strategy (Group 2).

Of the total number of individuals included in the initial study, the mean age was 61.7 ± 10.7 years and 74 patients were women (78.7%), without significant differences between both groups. The data about the number of consultations and imaging tests, the values of the blood tests, and the different times, are summarized in the Table 2.

**Table 2:** Differences between group 1 (successful conventional protocol) and group 2 (Failed conventional protocol + ^18^F-FCh PET-CT)

	Total *n* = 94	Group 1 *n* = 42	Group 2 *n* = 52
Number of pre-surgical Endocrinology consultations	7.33 ± 3.98	6.38 ± 3.66	8.35 ± 4.07 *
Number of pre-surgical General Surgery consultations	1.89 ± 1.35	1.69 ± 0.97	2.17 ± 1.62
Number of Complementary Imaging Tests	3.11 ± 1.39	2.31 ± 2.29	3.75 ± 1.38 *
Ca at diagnosis (mg/dL)	11.49 ± 0.73	11.71 ± 0.80	11.33 ± 0.63 *
P at diagnosis (mg/dL)	2.64 ± 0.45	2.66 ± 0.36	2.58 ± 0.53
PTH at diagnosis (pg/mL)	184.28 ± 116.57	178.41 ± 67.46	189.00 ± 145.06
Difference between PTH pre- and post-excision of the adenoma (%)	33.65 ± 25.57	28.52 ± 29.85	40.55 ± 30.42
Ca (mg/dL) 12 months post-operative	9.22 ± 1.34	9.40 ± 0.44	9.11 ± 1.67
Duration of intervention (min)	44.59 ± 29.68	43.17 ± 33.34	46.11 ± 27.02
Interval 1 st visit– surgical intervention (days)	793.74 ± 524.26	595.24 ± 325.11	948,78 ± 598.28 *

*p < 0.05

Although the standard diagnostic protocol was followed for most patients, in some cases, one of the tests could not be performed for various technical reasons, so the ^18^F-FCh PET-CT was requested. Since this study aims to show the reality of the process experienced by all patients, it was considered appropriate not to exclude these cases. 85 patients (90.4%) underwent cervical ultrasound, 8 patients (8.7%) underwent a cervical CT (not included in the standard protocol), 89 patients (94.7%) underwent ^99m^Tc-MIBI SPECT/scintigraphy, and 52 patients (55.3%) underwent ^18^F-FCh PET/TC.

Among the entire sample, there were no complications, except in only one patient in group 2 who presented transient dysphonia. To evaluate the results of the surgery, the differences between intraoperative PTH values ​​before and after excision and serum calcium preoperatively and after one year of follow-up were seen (Table 2).

Of the 94 cases included in the study, 52 ultimately underwent a ^18^F-FCh PET-CT, meaning that 55.3% of the patients included in the study obtained a failed or inconclusive result with the conventional diagnostic strategy (Table 3). All the ^18^F-FCh PET-CT performed were positive for the location of the adenoma.

**Table 3:** Differences in the economic assessment of the costs of the diagnostic process for location of parathyroid adenomas, using the conventional protocol (Cervical ultrasound + 99mTc-Scintigraphy/SPECT ± 18 F-FCh PET-CT) vs. the use of 18 F-FCh PET-CT as the only first-line technique

Conventional strategy unsuccessful (%)	55.3
Mean Price (± SD) of one patient for conventional strategy successful (€)	984.00 ± 325.62
Mean Price (± SD) of one patient for conventional strategy failed (€)	2,345.01 ± 520.72
Real costs of our 94 patients with conventional strategy (€)	162,677.34
Mean value (± SD) of real costs for each of our 94 patients (€)	1,730.61 ± 811.16
Costs of one patient with our ideal protocol (€)	1,399.77
Costs of 94 patients with our ideal protocol (€)	131,578.38
Savings for our hospital if the ideal proposed protocol had been used (€)	31,098.96

The cost of the diagnostic process was calculated by multiplying the price of each diagnostic test by the number of times it was performed. The different costs results are summarized in Table 3.

## Discussion

The two groups studied were homogeneous in terms of age and sex, with data similar to those reported by Ballester et al. [17] who carried out a prospective cohort study in which they included 34 patients with a diagnosis of PHPT with negative ^99m^Tc-MIBI scintigraphy/SPECT who underwent a ^18^F-FCh PET-CT; and were compared with the control group, in which the conventional diagnostic strategy without ^18^F-FCh PET-CT was positive.

In our series, there were also no statistically significant differences in the initial values ​​of PTH and P, although there were significant differences in the initial values ​​of Ca, which could suggest that the conventional diagnostic strategy could be more effective the higher the initial value of calcemia, and the more useful the ^18^F-FCh PET-CT in cases with lower calcemia. This partially coincides with Ballester et al. [17] who found statistically significant differences between groups in calcemia and PTH values ​​at diagnosis. Ballester et al. agree with us that the calcemia at diagnosis was lower in the group with a negative conventional diagnosis, mentioning the possible role of calcemia in the success of the diagnostic tests and the possible development of markers of negativity of the ^99m^Tc-MIBI scintigraphy/SPECT, which could be applied in decision making and choosing the test with the highest diagnostic yield [17].

Our population has a mean age of 61.7 ± 10.7 years and is made up mostly of women (78.7%); this coincides with the global distribution of PHPT, being much more frequent in women (the prevalence among women being 233 per 100,000 inhabitants, compared to 85 cases per 100,000 inhabitants in men) over 45 years of age [18]. Neither age nor sex influenced the positivity of the diagnostic tests.

Regarding the sensitivity of the diagnostic tests in our sample, the conventional diagnostic strategy obtained a sensitivity for the diagnosis of localization of 44.68% (42 positive cases out of 94 total), while the ^18^F-FCh PET-CT obtained a sensitivity of 100% (52 positive cases out of 52 patients who underwent the test). These data are close to the referred by Chakrabarty N et al. [2]who reported the sensitivity of the conventional strategy and the ^18^F-FCh PET-CT of 51.9–94.1% and 92-98.7% respectively, obtained from the latest review on the subject that includes studies carried out between the years 2021 and 2024 ^2^. The percentage of failed diagnoses with the two traditional tests in the study carried out by Mandic et al. [9] was 43% of the cases, with ^18^F-FCh PET-CT being used later in these cases and achieving a parathyroid adenoma detection rate of 95%. This study included a total of 83 patients with a sex distribution similar to ours (84.3% women) and an age of the sample studied like ours (57.2 years). It differs from our study in that it not only included single adenomas, but a total of 98 glands were studied in the total of 83 patients included in the study [9].

Despite the greater definition of the ^18^F-FCh PET-CT scan and the greater precision of the location, no statistical differences were found in the duration of the intervention. This data is mentioned by Rep et al. [19]who compared a group of 234 patients operated on for parathyroid adenoma after diagnosis of location with ^99m^Tc-sestamibi SPECT-CT and a second group of 163 patients operated on for parathyroid adenoma after diagnosis of location with ^18^F-FCh PET-CT. They concluded that there were no statistically significant differences between the duration of the intervention in the two groups. In addition, they mention that given the greater diagnostic accuracy of ^18^F-FCh PET-CT, intraoperative determination of PTH could be dispensed, which could reduce the duration of the intervention by up to 41%, also reducing the cost of the diagnostic and therapeutic process [19].

Regarding postoperative complications, they are very rare in the intervention for this pathology and in our study only one patient in group 2 presented transient dysphonia, which was not statistically significant. However, in the work of Rep et al. [19]it was concluded that there were differences between the group diagnosed with ^99m^Tc-MIBI scintigraphy/SPECT and the group diagnosed with ^18^F-FCh PET-CT. These differences were found in all the complications studied as a whole, but when studied individually, statistically significant differences were only found in reintervention, which was higher in patients diagnosed with ^99m^Tc-MIBI scintigraphy/SPECT (12.8%) compared to those diagnosed with ^18^F-FCh PET-CT (1.8%). No differences were found in tingling, Chvöstek’s sign, and hungry bone syndrome. Other complications such as discomfort, hoarseness, hypocalcemia, or hematoma were not studied because they were not described in one of the two groups. Furthermore, Rep et al. [19] emphasize the duration of each test and refers to their experience, in which a day can include the performance of twelve ^18^F-FCh PET-CT scintigraphies, compared to only three ^99m^Tc-MIBI scintigraphies/SPECT, so that, if ^18^F-FCh PET-CT were applied preferentially, the waiting list could probably be reduced, at least for the diagnosis of location.

There were no differences between our two groups in the results: we found no significant differences between our two groups regarding the intraoperative decrease in PTH, nor in terms of serum calcium levels one year after the intervention. These results are similar to those reported by Ballester et al. [17] who reported that no differences were found in the criterion of cure, which consisted of a > 50% decrease in PTH values ​​10 min after excision of the suspicious gland compared to the values ​​prior to excision, and which are basically like those reported by Rep et al. [19]so, from the clinical point of view, making a diagnosis of location with ^18^F-FCh PET-CT does not provide significant improvement over cases in which a positive diagnosis has been achieved with the traditional method.

The results show that the process was clearly more expensive in patients who needed an ^18^F-FCh PET-CT than in those who were initially diagnosed with the conventional protocol. This is in line with expectations, since in these cases, in addition to including the price of the new examination, a greater number of conventional examinations were performed, there were more consultations, and more time was spent on the diagnostic process.

From the patient’s personal point of view, they prefer a method that achieves the highest percentage of diagnostic success on its own, because it significantly reduces the number of visits to the hospital and the number of tests performed, reducing the duration of the pre-surgical process, and thus achieving resolution of the problem much sooner. This problem is reflected in most studies carried out, in which second-line ^18^F-FCh PET-CT has been performed once the conventional diagnosis is negative, doubtful or discordant, which in all cases entails a greater number of complementary examinations and a longer time until the location diagnosis.

From the economic point of view, given the number of doubtful results present in the conventional protocol, which require the performance of complementary second-line methods in many patients, in our opinion it is much more efficient to start directly with the use of ^18^F-FCh PET-CT as the sole first-line diagnostic method, as referred by Yap et al. [14]. Although it is a more expensive process when analyzed patient by patient, it is cheaper when all patients and the expenses they generate are studied globally together. And the differences would probably increase if the cost of medication for the hypercalcemia (cinacalcet, bifosfonates.) maintained for a longer period until the surgical intervention is performed was added, although this parameter has not been studied by us.

Regarding the overall cost of the process, Yap et al. [14]who carried out the first cost-effectiveness study, concluded that the total cost of the treatment (which includes the diagnostic process and etiological treatment), carried out by ^18^F-FCh PET-CT was $11,245. The cost of the process with diagnosis based in ultrasound and ^99m^Tc-MIBI scintigraphy was $9,901 and $10,350 respectively; these two were rejected because they cost more than the complete therapeutic process with 4D CT ($9,677) and provided less health benefits. Therefore, the cost-effectiveness of 4D CT was compared with ^18^F-FCh PET-CT. Although the cost was higher for diagnosis using ^18^F-FCh PET-CT, this diagnostic test accumulated greater utility in health, so it was concluded that the therapeutic process was more cost-effective clinically with ^18^F-FCh PET-CT than with 4D CT. These findings support our results. It must be considered that this study was carried out a few years ago, when the cost of diagnostic tests (especially ^18^F-FCh PET-CT) was $2,096, while currently, in our setting it is €1,026.66. Furthermore, it was a study carried out in the United States, so prices may vary from those in Europe. Other studies, including Zarei et al. [7]do not comment on the absolute cost of each diagnostic strategy, but they do mention that the ^18^F-FCh PET-CT strategy is more expensive than the conventional strategy. We agree that this strategy is indeed more expensive if we consider one patient, but it is cheaper if we consider all the patients operated on, as our study shows.

There is an interesting study performed by Mossel et al. [12] that compares on a theoretical basis the differences between two diagnostic protocols, using ^18^F-FCh PET-CT as the first and only line, or the traditional one. It assesses the usefulness of both and concludes that they are similar. Using probabilistic models, it calculates that only the ^18^F-FCh PET-CT model would be profitable if the price of the examination were lower, or if the sensitivity of the MIBI were reduced in the setting in which it is being used. This is a theoretical study with economic values ​​relatives to Netherlands, but in any case, it does not consider the use of ^18^F-FCh PET-CT as the first and only line to be more expensive.

Along the same lines as the previous study, Mamou et al. [13] conducted a comprehensive study of the economic cost of surgery and the use of different diagnostic imaging techniques. The values ​​are different from ours, even though they belong to nearby European countries: Cervical ultrasound + ^99m^Tc-MIBI scintigraphy/SPECT = 306,6 € ^18^F-FCh PET-CT = 789,5 €. They found the Cervical ultrasound + ^99m^Tc-MIBI scintigraphy/SPECT system to be the most cost-effective protocol, considering the performance of ^18^F-FCh PET-CT only if the previous protocol is negative ^18^F-FCh PET-CT alone as a first-line diagnostic procedure becomes more effective as the number of examinations increases, which also reduces the price. This also depends on the percentage of positive MIBI diagnoses at each center [13].

Most studies [12–14, 17] agree that the results of surgery are not affected by the diagnostic method used, as we have also verified. However, there is a reduction in the time required for diagnosis, which impacts the improvement in quality perceived by patients.

Our study has the strength of having taken the last 100 consecutive cases, without applying any bias, and taking the real data of the diagnostic process and its cost. As limitations of the work, we can mention that it is a retrospective study, and that the protocol proposed as an alternative is not indicative of a real population, but of an ideal population. However, the results would justify the continuation of the research, possibly studying 100 consecutive cases in which the proposed ideal protocol was applied.

In our setting, the overall cost of diagnosing the location of parathyroid adenoma in a group of patients with primary hyperparathyroidism is lower with a diagnostic strategy using ^18^F-FCh PET-CT as a first line only test than with conventional diagnosis with ^99m^Tc-MIBI scintigraphy/SPECT and cervical ultrasound.

The conclusion can be that the use of ^18^F-FCh PET-CT as a first-line diagnostic test for location of parathyroid adenomas is very effective in terms of results of surgery, reduces the duration of the whole process and its cost-effectiveness depends largely on the price of the examinations, depending on the country and health system, and on availability at each center. In Spain, when available in the hospital, its use as a first line imaging test would be justified.

## Data Availability

The data that support the findings of this study are not openly available due to reasons of sensitivity and are available from the corresponding author upon reasonable request.

## References

[1] Palermo A, Tabacco G, Makras P, Zavatta G, Trimboli P, Castellano E et al (2024) Primary hyperparathyroidism: from guidelines to outpatient clinic. Rev Endocr Metab Disord. Available from: 10.1007/s11154-024-09899-5 10.1007/s11154-024-09899-5 39162944

[2] Chakrabarty N, Mahajan A, Basu S, D’Cruz AK (2024) Imaging recommendations for diagnosis and management of primary parathyroid pathologies: A comprehensive review. Cancers 16(14):2593. 10.3390/cancers16142593 39061231 10.3390/cancers16142593 PMC11274996

[3] Fiz F, Bottoni G, Massollo M, Trimboli P, Catrambone U, Bacigalupo L et al (2023) [18F]F-Choline PET/CT and 4D-CT in the evaluation of primary hyperparathyroidism: rivals or allies? Q J Nucl Med Mol Imaging 67(2):130–137. 10.23736/S1824-4785.23.03514-8 37232932 10.23736/S1824-4785.23.03514-8

[4] Quak E, Lheureux S, Reznik Y, Bardet S, Aide N (2013) F^18^-choline, a novel PET tracer for parathyroid adenoma? J Clin Endocrinol Metab 98(8):3111–3112. 10.1210/jc.2013-2084 23788686 10.1210/jc.2013-2084

[5] Ferrari C, Santo G, Mammucci P, Pisani AR, Sardaro A, Rubini G (2021) Diagnostic value of choline PET in the preoperative localization of hyperfunctioning parathyroid gland(s): A comprehensive overview. Biomedicines 9(3):1–18. 10.3390/biomedicines9030231 10.3390/biomedicines9030231 PMC7996619 33669104

[6] Piccardo A, Trimboli P, Rutigliani M, Puntoni M, Foppiani L, Bacigalupo L et al (2019) Additional value of integrated 18 F-choline PET/4D contrast-enhanced CT in the localization of hyperfunctioning parathyroid glands and correlation with molecular profile. Eur J Nucl Med Mol Imaging 46(3):766–775. 10.1007/s00259-018-4147-4 30219964 10.1007/s00259-018-4147-4

[7] Zarei A, Karthik S, Chowdhury FU, Patel CN, Scarsbrook AF, Vaidyanathan S (2022) Multimodality imaging in primary hyperparathyroidism. Clin Radiol. 77(6):e401-16. 10.1016/j.crad.2022.02.018 35393101 10.1016/j.crad.2022.02.018

[8] Quak E, Cavarec M, Ciappuccini R, Girault G, Rouzier R, Lequesne J (2023) Detection, resection and cure: a systematic review and meta-analysis of 18F-choline PET in primary hyperparathyroidism. Q J Nucl Med Mol Imaging 67(2):122–129. 10.23736/S1824-4785.23.03512-4 36756935 10.23736/S1824-4785.23.03512-4

[9] Mandic A, Kraljevic I, Polovina TS, Tomsic KZ, Dusek T, Balasko A, Solak M, Kastelan D (2024) Diagnostic performance of 99mTc-Sestamibi SPECT/CT and 18F-Choline PET/CT in locating hyperfunctioning parathyroid glands in primary hyperparathyroidism patients. Exp Clin Endocrinol Diabetes 132(04):216–220. 10.1055/a-2262-9249 38320618 10.1055/a-2262-9249

[10] Petranovic Ovcaricek P, Giovanella L, Carrio-Gasset I et al (2021) The EANM Parctice guidelines for parathyroid imaging. Eur J Nucl Med Mol Imaging 48:2801–2822. 10.1007/s00259-021-05334-y 33839893 10.1007/s00259-021-05334-y PMC8263421

[11] Petranović Ovčariček P, Giovanella L, Hindie E, Huellner MW, Talbot JN, Verburg FA (2022) An essential practice summary of the new EANM guidelines for parathyroid imaging. Q J Nucl Med Mol Imaging 66:93–103. 10.23736/S1824-4785.22.03427-6 35166093 10.23736/S1824-4785.22.03427-6

[12] Mossel S, Saing S, Appelman-Dijkstra N, Quak E, Schepers A, Smit F, de Geus-Oei L-F, Vriens D (2024) Cost-effectiveness of one-stop-shop [18F]Fluorocholine PET/CT to localise parathyroid adenomas in patients suffering from primary hyperparathyroidism. Eur J Nucl Med Mol Imaging 51(12):3585–3595. 10.1007/s00259-024-06771-1 38837058 10.1007/s00259-024-06771-1 PMC11457719

[13] Mamou A, Chkair S, Gilly O, Maimoun L, Mamou Y, Sheppard SC, Kotzki PO, Lallemant B, Boudous V (2025) Economic evaluation of [18F]fluorocholine PET/CT in pre operative assessment of hyperfunctional parathyroids in primary hyperparathyroidism: a cost effectiveness análisis. EJNMMI Rep 911. 10.1186/s41824-025-00244-w 10.1186/s41824-025-00244-w PMC11958853 40164868

[14] Yap A, Hope TA, Graves CE, Kluijfhout W, Shen WT, Gosnell JE et al (2022) A cost-utility analysis of 18F-fluorocholine–positron emission tomography imaging for localizing primary hyperparathyroidism in the united States. Surgery 171(1):55–62. 10.1016/j.surg.2021.03.075 34340823 10.1016/j.surg.2021.03.075

[15] Servicio de Información sobre la Gestión Económica (SIGE) (2023) Hospital Clínico Universitario de Valencia. Sistema de Información Económica (SIE)

[16] Ley de tasas de la Generalitat Valenciana (2027) Diario Oficial de la Generalitat Valenciana (DOGV), nº. Ley 20:86–115 Texto consolidado para 2022. 28 de diciembre de 2017

[17] Ballester Vázquez E, Pérez García JI, López Mora DA, Galán Martínez C, Pareja Nieto E, Clos Enríquez M et al (2020) Identification of occult adenomas in primary hyperparathyroidism with 18F-fluorocholine PET/CT. Cir Esp 98(7):395–402 32115188 10.1016/j.ciresp.2020.01.003

[18] Minisola S, Arnold A, Belaya Z, Brandi ML, Clarke BL, Hannan FM et al (2022) Epidemiology, pathophysiology, and genetics of primary hyperparathyroidism. J Bone Min Res 37(11):2315–2329 10.1002/jbmr.4665 PMC10092691 36245271

[19] Rep S, Sirca K, Lezaic EM, Zaletel K, Hocevar M, Lezaic L (2024) 18F]fluorocholine PET vs. [99mTc]sestamibi scintigraphy for detection and localization of hyperfunctioning parathyroid glands in patients with primary hyperparathyroidism: outcomes and resource efficiency. Radiol Oncol 58(4):486–493 39608010 10.2478/raon-2024-0058 PMC11604255

